# Supplementary material for: Prevalence of the Puumala orthohantavirus Strains in the Pre-Kama Area of the Republic of Tatarstan, Russia
Source: Pathogens. 2020 Jul 6;9(7):540. doi: 10.3390/pathogens9070540 (PMC7400482; doi:10.3390/pathogens9070540)
Supplement: Supplementary file 1 [file pathogens-09-00540-s001.pdf]

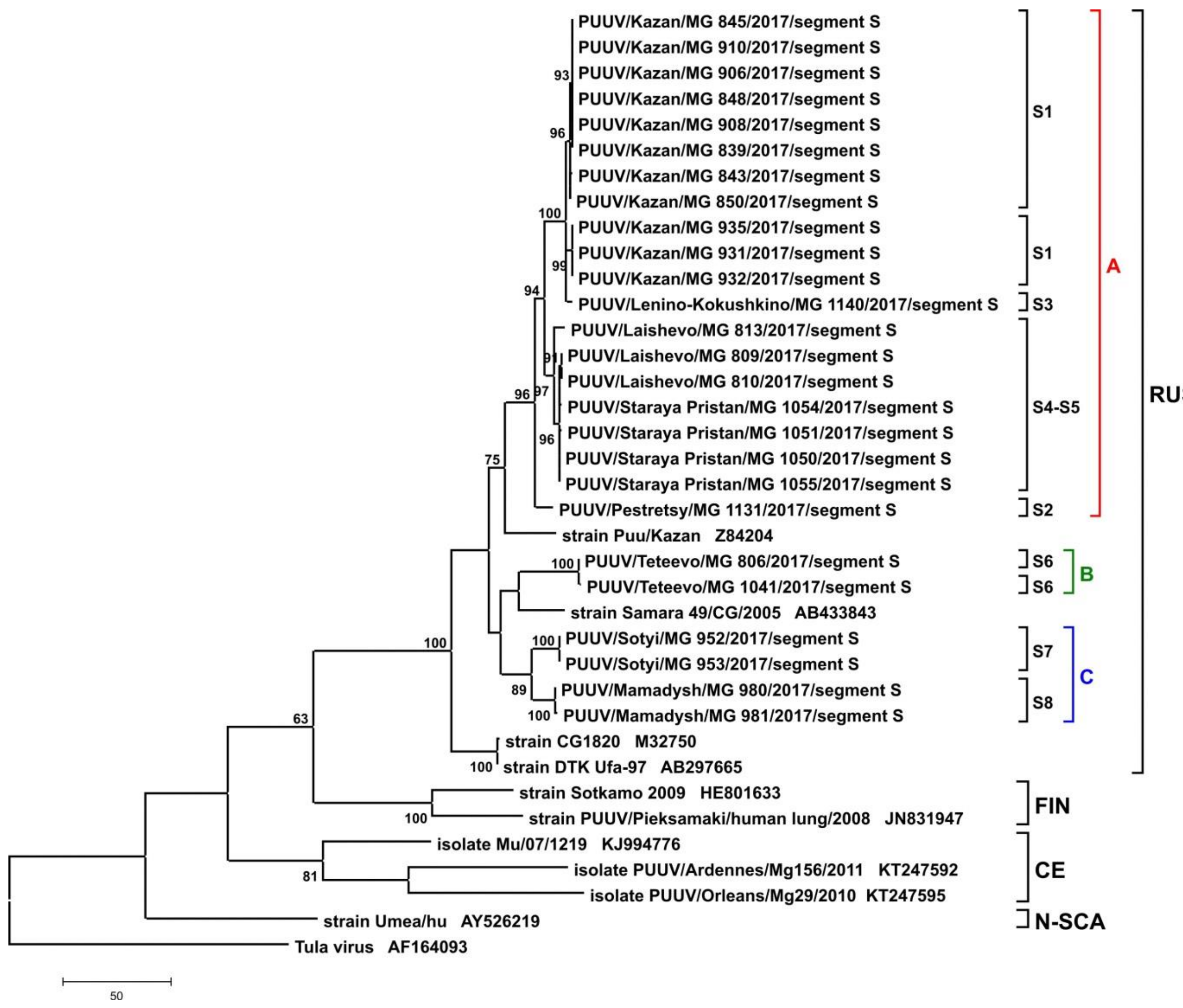

Figure S1. Phylogenetic tree for the partial S segment of "RT-2017" (nt 240-1296 based on GenBank sequence Z84204) constructed by Maximum Parsimony method. Bootstrap values were calculated for 1000 replicates; only values greater than 70% are shown.

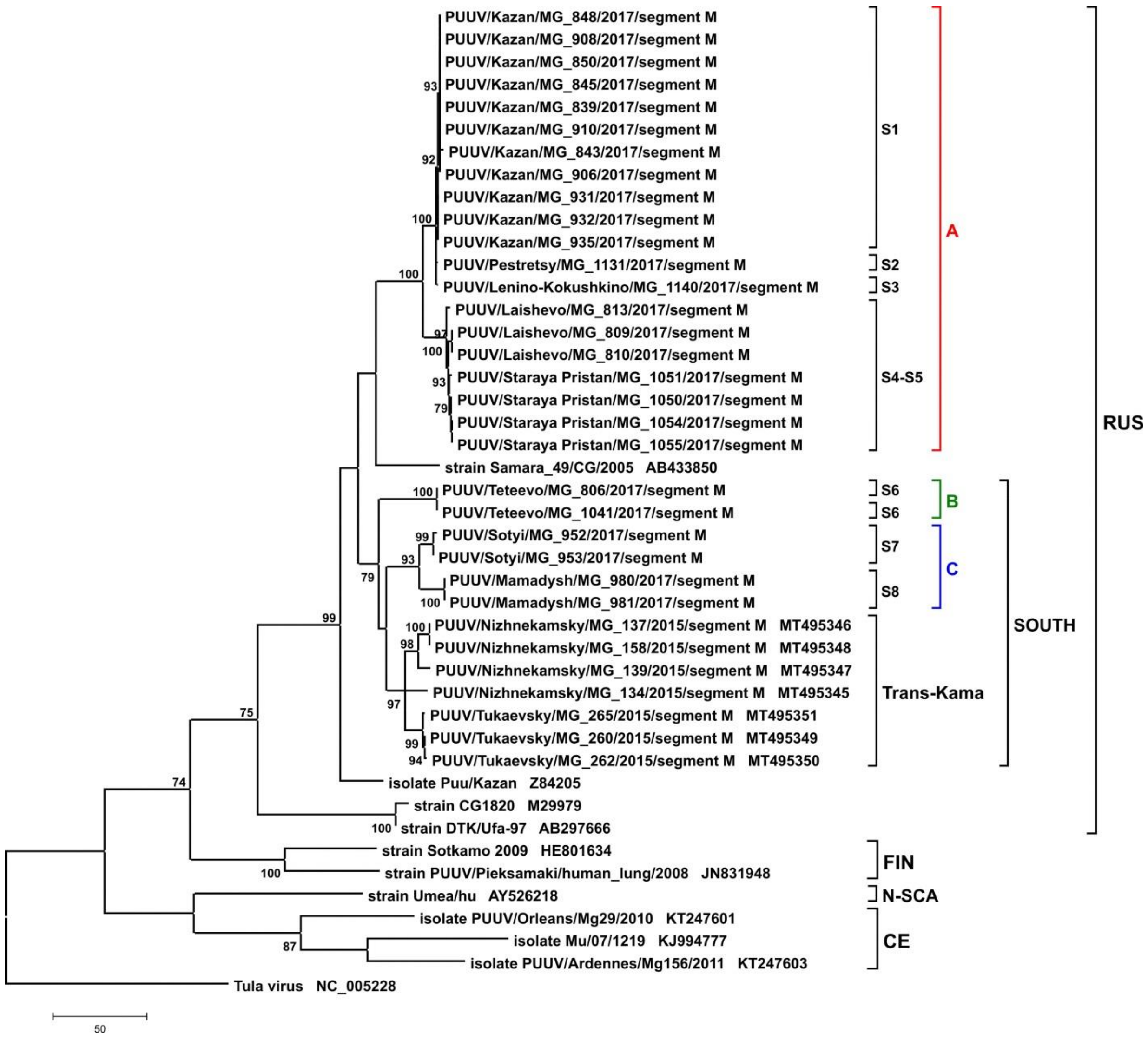

Figure S2. Phylogenetic tree for the partial M segment of "RT-2017" (nt 1499-2512 based on GenBank sequence Z84205) constructed by Maximum Parsimony method. Bootstrap values were calculated for 1000 replicates; only values greater than 70% are shown.

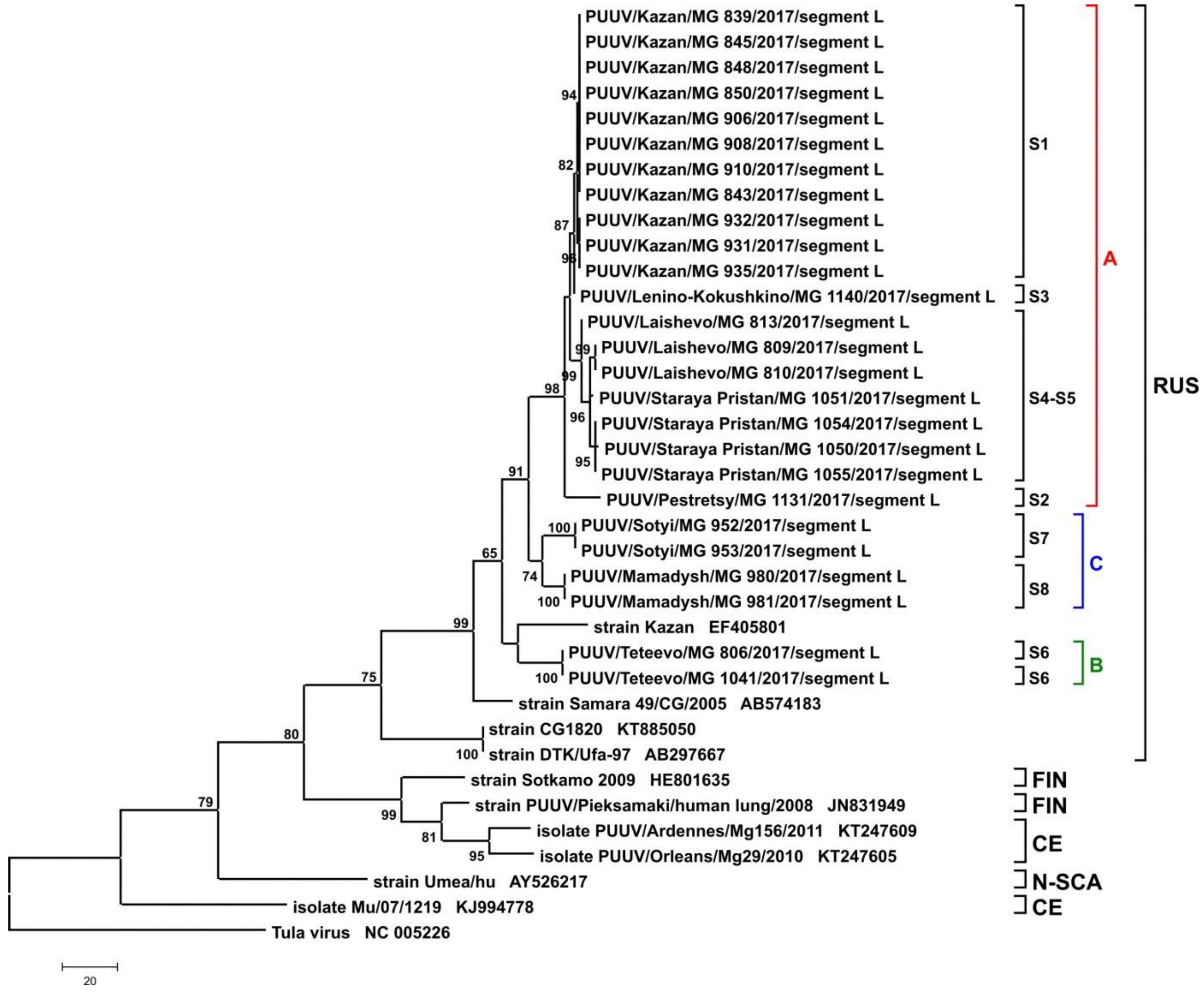

Figure S3. Phylogenetic tree for the partial L segment of "RT-2017" (nt 958-1622 based on GenBank sequence EF405801) constructed by Maximum Parsimony method. Bootstrap values were calculated for 1000 replicates; only values greater than 70% are shown.

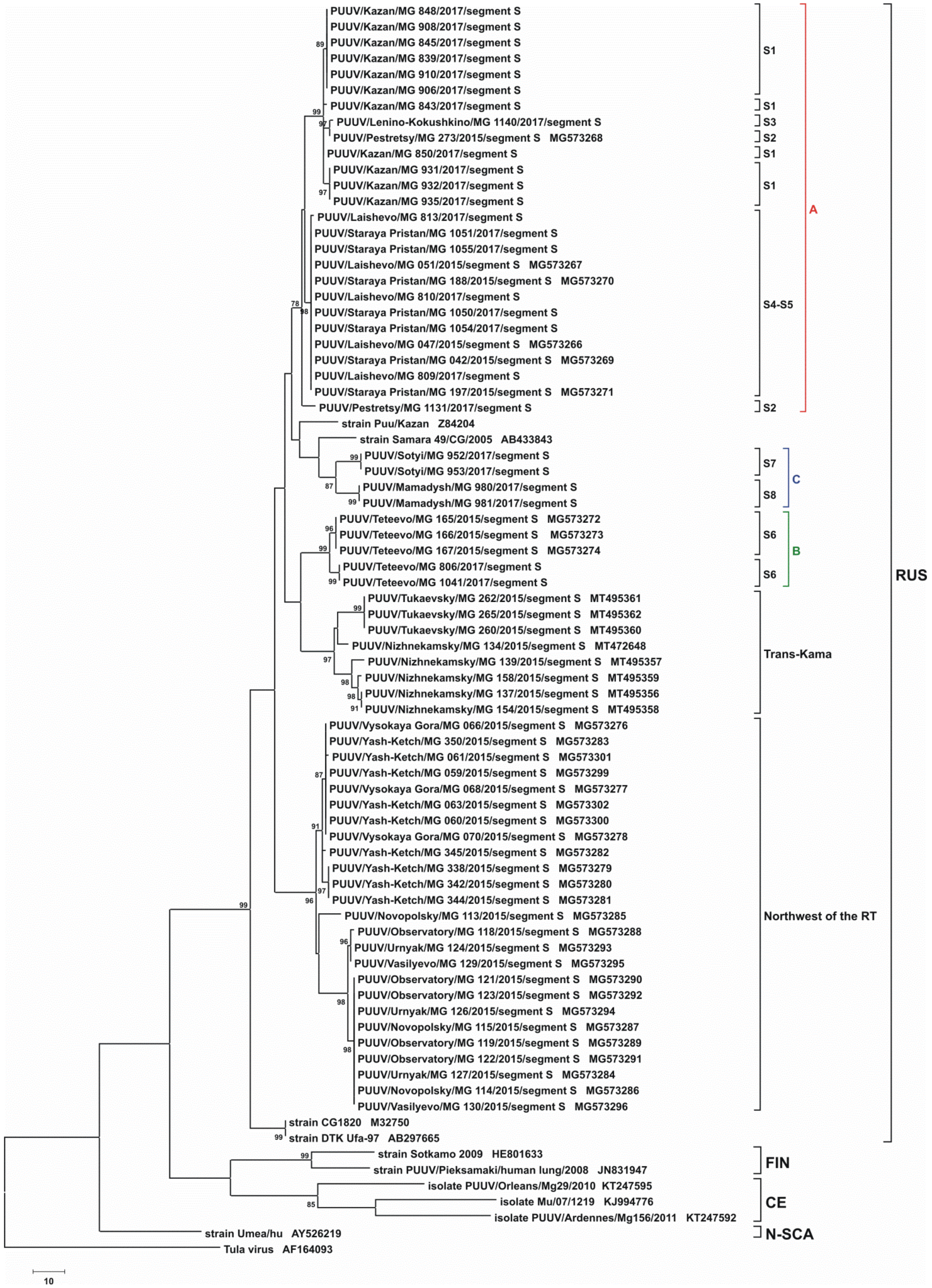

Figure S4. Phylogenetic tree for the partial S segment of PUUV strains from RT (nt 242-805 based on GenBank sequence Z84204) constructed by Maximum Parsimony method. Bootstrap values were calculated for 1000 replicates; only values greater than 70% are shown.
